# BiOBr/BiOI Photocatalyst Based on Fly Ash Cenospheres with Improved Photocatalytic Performance

**DOI:** 10.3390/molecules21050666

**Published:** 2016-05-19

**Authors:** Li Lin, Manhong Huang, Donghui Chen

**Affiliations:** 1 School of Chemical and Environmental Engineering, Hunan City University, Yiyang 413000, China; 2 School of Environmental Science and Engineering, Donghua University, Shanghai 201620, China; egghmh@163.com; 3 Shanghai Institute of Technology, Shanghai 200235, China

**Keywords:** BiOBr/BiOI, fly-ash cenospheres, photocatalytic activity, pH value

## Abstract

A series of BiOBr/BiOI photocatalysts supported on fly-ash cenospheres (FACs) were successfully prepared via a facile one-pot alcoholysis method. The as-prepared samples were characterized by X-ray diffraction (XRD), scanning electron microscopy (SEM), X-ray photoelectron spectrometer (XPS) and UV-visible diffuse reflectance spectroscopy (DRS). The results indicate that pH value plays a critical role in BiOBr/BiOI loading. Based on the photodegradation tests under visible light irradiation (blue LED irradiation), the photocatalytic property of BiOBr/BiOI/FACs photocatalysts obtained under alkaline conditions is superior to that prepared under neutral or acidic conditions, and higher than those of BiOB/FACs and BiOI//FACs. The improved photocatalytic performance of BiOBr/BiOI/FACs can be attributed to more BiOBr/BiOI loaded on the surface of FACs and the efficient photogenerated electron-hole separation.

## 1. Introduction

Heterogeneous photocatalysis is considered an environmentally friendly technique for the elimination of organic contaminants, and as such, has attracted much attention over the past 30 years [1,2,3]. The most attractive photocatalyst, TiO_2_ (with a band gap of 3.2 eV), can only be activated by UV light irradiation (which accounts for just 4% of the solar irradiation) which hinders its practical application [4]. Therefore, many photocatalytic materials, such as single bismuth oxyhalides (BiOX, X = Cl, Br, I) [5], and their derivatives BiOCl_x_Br_1−x_ [6] and BiOBr_x_I_1−x_ [7], have been developed in recent years as prospective photocatalysts. Especially, BiOBr and BiOI, with their narrow band gaps of 2.60 eV and 1.71 eV, can be directly excited by visible light. It is worthwhile noting that these particle-photocatalysts will have restricted applications because of their poor usage of light, hard recycling and low removal efficiency for surface pollutants in the sea or lakes, such as floating oil and algae. Coating the particles onto a support has been considered to be one of the ideal methods for solving this problem in recent years [8,9,10,11,12]. As a kind of by-product produced during the combustion of coal in thermal power plants, the use of fly-ash cenospheres (FACs) as supports to coat different photocatalysts have been reported due to the carriers’ advantages of low cost, fine particle size, non-toxicity, low density, excellent mechanical properties and ample availability from coal fired power plants [13,14]. More importantly, floatable photocatalysts prepared based on FACs can be excited by more solar light on the surface of water. This is favorable to improve the usage of solar light and the pollutant removal efficiency, especially for surface pollutants in the sea or lakes. Therefore, it is reasonable to expect that bismuth oxyhalides (BiOX, X = Cl, Br, I) and their composites supported on the surface of FACs may also exhibit unique properties. In our preliminary experiments [15,16], three-dimensional (3D) BiOBr/BiOI hierarchical microsphere photocatalysts were successfully synthesized via a facile one-pot solvothermal method. A 50% BiOBr/BiOI composite showed the highest photocatalytic removal efficiency for rhodamine B (RhB) and tetracycline (TC), and a hierarchical microsphere BiOBr was fabricated on the surface of FACs by the same method and also showed high photocatalytic activity. However, the solvothermal process must be carried out in a Teflon-lined stainless steel autoclave. This inspired us to explore simpler approaches for the fabrication of BiOI/BiOBr/FACs with unique properties. To the best of our knowledge, there have been no reports of similar studies, so herein, for the first time, we report a facile and one-pot alcoholysis method to fabricate BiOI/BiOBr/FACs. It is found that pH value plays the vital factor for the loading of BiOI/BiOBr on the surface of FACs. The photocatalytic activity of the as-obtained novel photocatalysts was investigated by treating RhB aqueous solution under blue LED irradiation with a main wavelength at 450 nm which represents the main energy band of sunlight.

## 2. Results and Discussion

### 2.1. XRD Patterns

**Figure 1:**
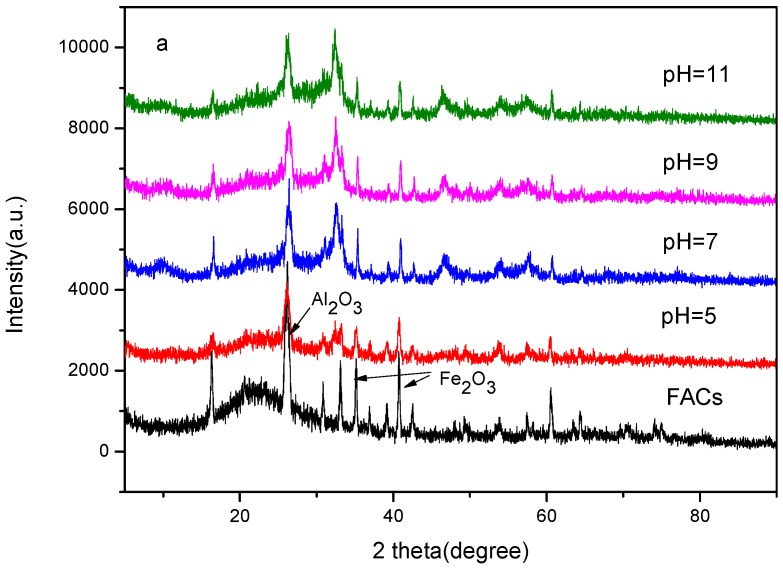

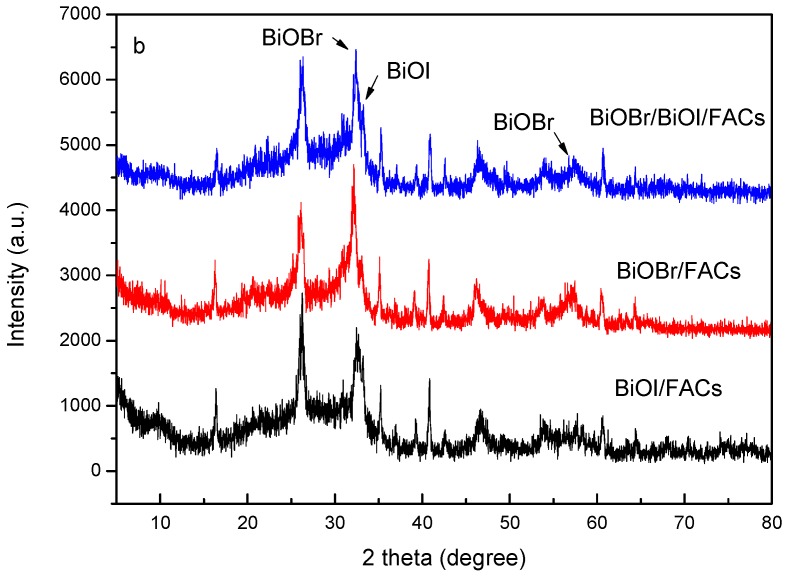
XRD patterns of the samples: (**a**): FACs and samples obtained at different pH values; (**b**): BiOBr/BiOI/FACs , BiOI/FACs and BiOBr/FACs obtained under the same conditions (pH = 9).

The XRD spectra of FACs and different pH series samples are shown in Figure 1a, where the X-ray diffraction pattern of all the products can be indexed as the tetragonal phase 50%BiOBr/BiOI [7,15,17]. For comparasion, the X-ray diffraction pattern of BiOBr/FACs and BiOI/FACs obtained at pH 9 are also shown in Figure 1b, where the corresponding diffraction peaks can be indexed to the tetragonal BiOI phase (JCPDS card No. 10-0445) and the tetragonal BiOBr phase (JCPDS card No. 09-0393), respectively. The results indicate that BiOBr/BiOI is successfully coated on the surface of FACs. In addition, according to the XRD spectra of FACs carriers, some SiO_2_, Fe_2_O_3_ and Al_2_O_3_ peaks are all clearly seen in low pH samples, because the FACs are aluminosilicate-based ceramic particles and the main components of FACs are siliceous oxide, ferrous oxide and aluminous oxide, *etc.* [18]. However, with increasing pH value, the intensity of the SiO_2_, Fe_2_O_3_ and Al_2_O_3_ diffraction peaks is weakened as more BiOBr/BiOI is loaded on the FACs surface. Simultaneously, although possessing a consistent characteristic, the inflection peak intensity of BiOBr/BiOI loaded at high pH values is increased relative to that at low pH value. This results suggests that the increase of pH value is beneficial for the loading of BiOBr/BiOI on FACs, which may be favourable for the photocatalytic ability.

### 2.2. SEM and EDS Observation

**Figure 2:**
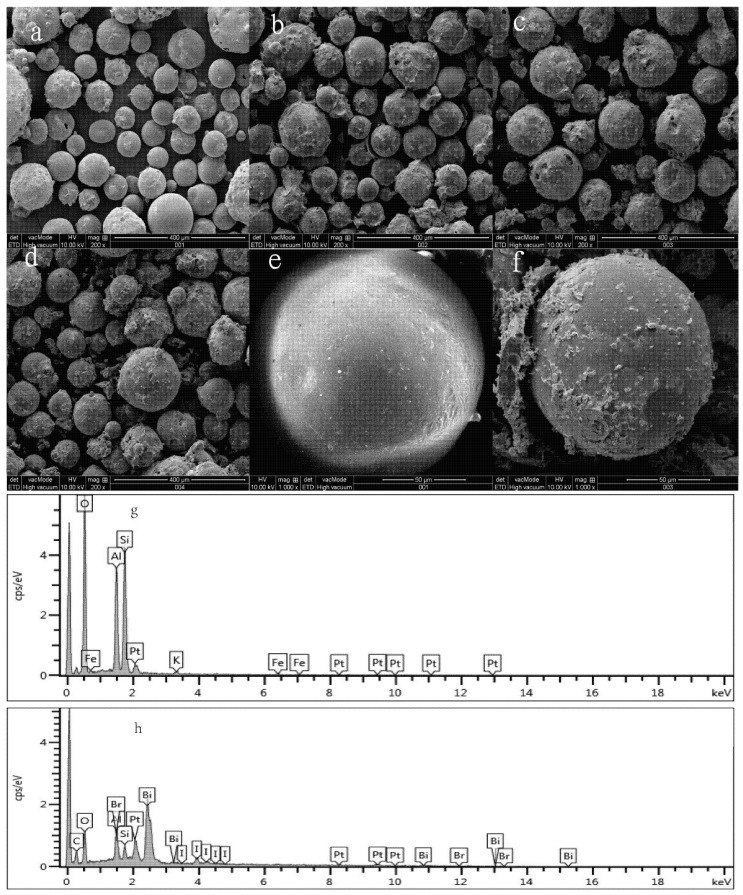
SEM images of a series of different pH samples: (**a**) pH = 5.0; (**b**) pH = 7.0; (**c**, **f**) pH = 9.0 (**d**) pH = 11.0; (**e**) the pure FACs; The EDS spectra of: (**g**) the supported materials FACs and (**h**) the loaded BiOBr/BiOI.

The SEM images of different pH series samples are shown in Figure 2. Figure 2a,e show that the FACs exhibit an essentially spherical and relatively uniform smooth-faced shape with a diameter of 100 μm.

Figure 2a–d show the SEM images of the products at low magnifications corresponding to as-obtained samples with alcoholysis pH values of 5.0, 7.0, 9.0 and 11.0, respectively. Figure 2a shows that few BiOBr/BiOI composites were coated on the FAC surface when the alcoholysis process was conducted under acidic conditions. In contrast, Figure 2b,d present the surface micrographs of the BiOBr/BiOI/FACs samples obtained under neutral and alkaline conditions. Different from that prepared under acidic conditions, the FACs surface is rough and loaded with a great deal of BiOBr/BiOI composites. Additionally, all these loaded BiOBr/BiOI composites presented thin films or irregular shapes. As seen from the high magnification image (Figure 2f) which presents a surface micrograph of a high pH sample, the rough surface of the FACs is useful for photocatalysis [19]. Figure 2g,h show representative EDS spectra of the FACs support and the loaded BiOBr/BiOI compounds, respectively. The EDS analysis illustrates that the major constituents for the supported materials are Fe, O, Si and Al. The peaks of Bi, Br, I and O are mainly generated by the BiOBr/BiOI. These results are consistent with the XRD data (Figure 1).

The proposed mechanism of BiOBr/BiOI/FACs composite synthesis is as follows: firstly, according to reaction (1), a coordination effect ocurrs between Bi^3+^, EG and a few H_2_O molecules; in this step the solution was transparent. However, in the following reaction (2), the OH^−^ ion caused the progress of the alcoholysis forming a coordination compound with the addition of ammonia, and the solution gradually became turbid when the solution went from acidic to alkaline conditions:

xBi^3+^ + zH_2_O + yEG → Bi_x_(EG)_y_(OH)_z_ + zH^+^ (1)

Bi_x_(EG)_y_(OH)_z_ + zOH^−^ + xBr^−^(I^−^) → xBiOBr(I) + yEG + zH_2_O (2)

The white sol-gel formed at higher pH could be easily adsorbed on the surface of FACs so that large number of photocatalyst precursor can be coated on them [20]. This is the key factor for the improvement of the photocatalytic properties under alkaline synthesis conditions. This mass increase of the coated BiOBr/BiOI has been verified by the SEM and XRD analysis.

### 2.3. DRS Analysis

**Figure 3:**
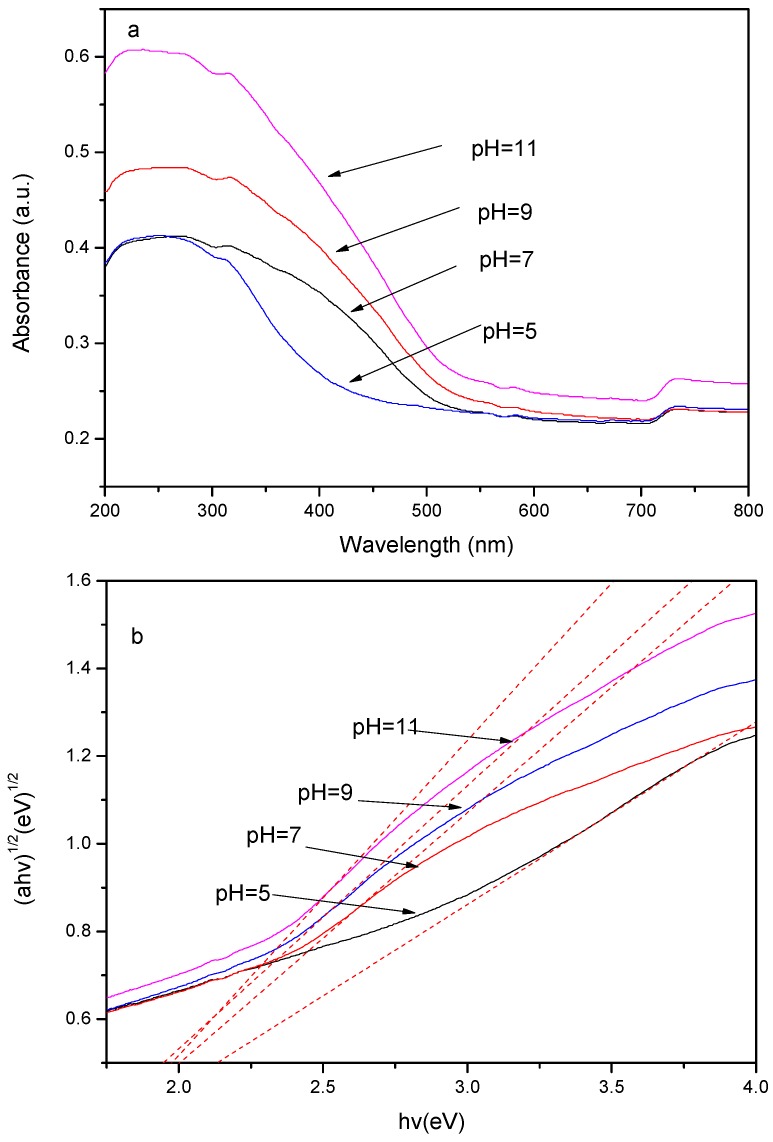
The UV-vis diffuse reflection spectra of a series of different pH samples (**a**) and plots of (α*h*γ)^1/2^ *vs.* photon energy (**b**).

The UV–Vis diffuse reflectance spectra of a series of different pH samples are shown in Figure 3. The results indicate that BiOBr/BiOI/FACs samples exhibit gradually increasing optical absorption in the visible light region of 450–550 nm as the alcoholysis pH value increases from 5.0 to 11.0. This intensified optical absorption could be attributed to more BiOBr/BiOI composites being loaded on the surface of FACs. The band gap energy of the as-synthesized samples could be calculated by the following formula [21,22]:

α*h*γ = A(*h*γ − Eg)^n/2^ (3)

where α, γ, Eg, and A are the absorption coefficient, light frequency, band gap, and a constant, respectively. The term n depends on the characteristics of the transition in a semiconductor, including direct transitions (*n* = 1) or indirect transitions (*n* = 4). As previous reports indicated that BiOX (X = Br, I) was an indirect band gap material [23], the band gap energy could be estimated from a plot of (α*h*γ)^1/2^ *vs.* the photon energy (*h*γ). The x-axis intercept of the tangent to the plot approaches the band gap energy of the sample. The band gap value of FACs samples is estimated to be 2.20 eV. The calculated result also demonstrates that the obtained BiOBr/BiOI/FACs composites have the similar band gap values of 1.94, 1.97, 1.98 and 2.01 eV, corresponding to alcoholysis pH values of 11.0, 9.0, 7.0 and 5.0, respectively. The result implies that the energy band gap of the photocatalysts has never obviously narrowed. In all, the series of supported photocatalysts, especially those obtained at high pH value, has potential application as visible light photocatalysts.

### 2.4. XPS Analysis

**Figure 4:**
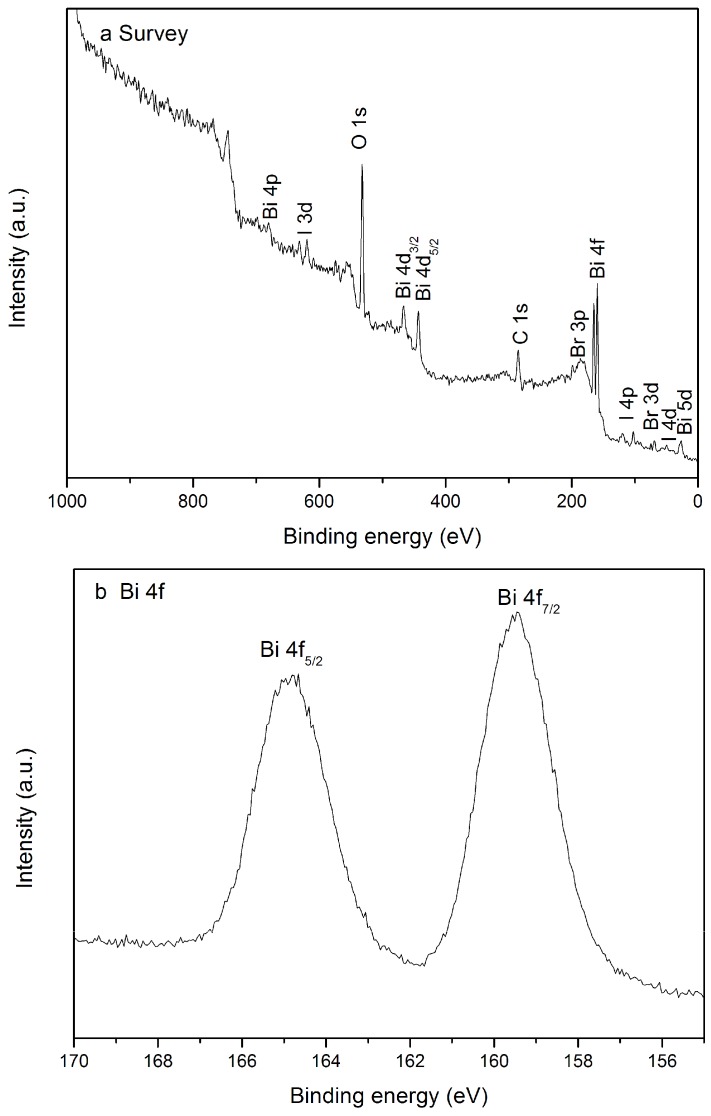

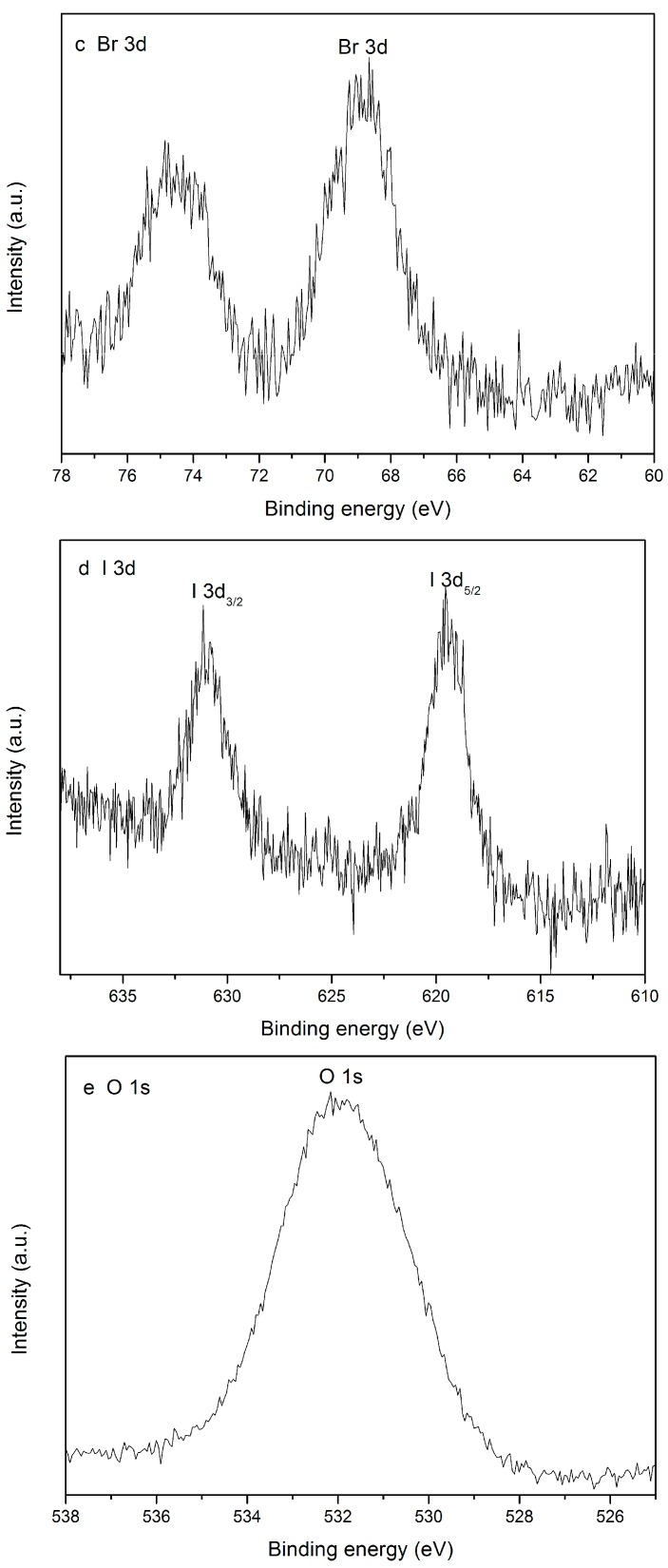
XPS spectra of as-prepared sample (pH = 9): (**a**) survey; (**b**) Bi 4f; (**c**) Br 3d; (**d**) I 3d and (**e**) O 1s.

The survey XPS spectra are shown in Figure 4. The results indicate that the Bi, Br, I and O elements are definitely detected in the as-prepared BiOBr/BiOI/FACs composites. The C 1s peak at 284.8 eV may originate from adventitious hydrocarbon contaminants in the XPS instrument analysis [24]. Figure 4b shows the high-resolution XPS spectrum of Bi 4f. The peaks with binding energy of 159.4 and 164.8 eV are separately ascribed to the Bi 4f_7/2_ and Bi 4f_5/2_, respectively, which is characteristic of the Bi^3+^ in the samples [25,26]. In Figure 4c, the peak located at 68.6 eV is assigned to the Br 3d peak, corresponding to the characteristics of the Br^−^ in the as-prepared materials [25]. The XPS I 3d spectrum in Figure 4d shows two peaks at 631.0 and 619.5 eV, which can be ascribed to I 3d_3/2_ and I 3d_5/2_, respectively [24,25]. Besides, Figure 4e shows the high-resolution XPS spectrum for the O 1s region that can be fitted to two peaks. The main peak at 530.4 eV is attributed to the Bi-O bonds in (BiO)_2_^2+^ slabs of the BiOX layered structure, and the peak at 532.1 eV is assigned to the hydroxyl groups on the surface [27,28]. The XPS quantification report of the as-synthesized BiOBr/BiOI/FACs samples obtained at pH 9 is illustrated in Table 1. According to the XPS analysis results, the I^−^ atomic concentration is lower than the theoretical value, which may be induced by the the loss of I^−^ in the open oven system at 160 °C.

### 2.5. Photocatalytic Evaluation

**Figure 5:**
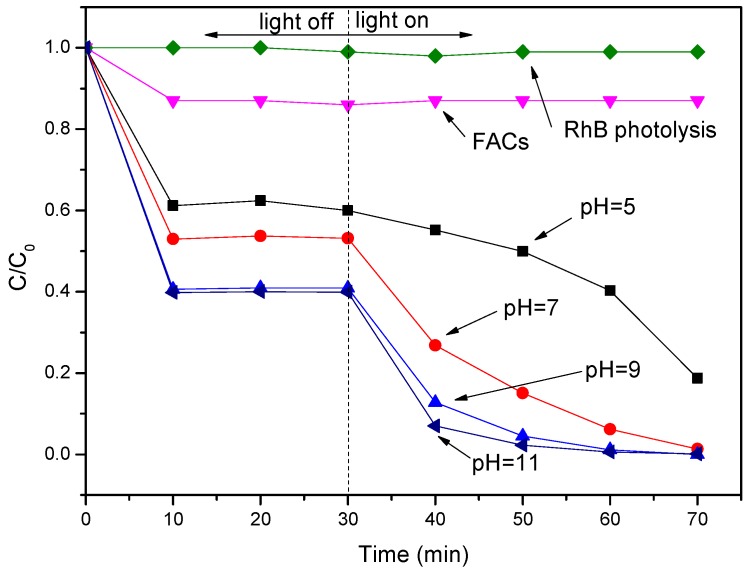
The degradation ratio-time curves of RhB over BiOBr/BiOI/FACs obtained at different pH values.

**Figure 6:**
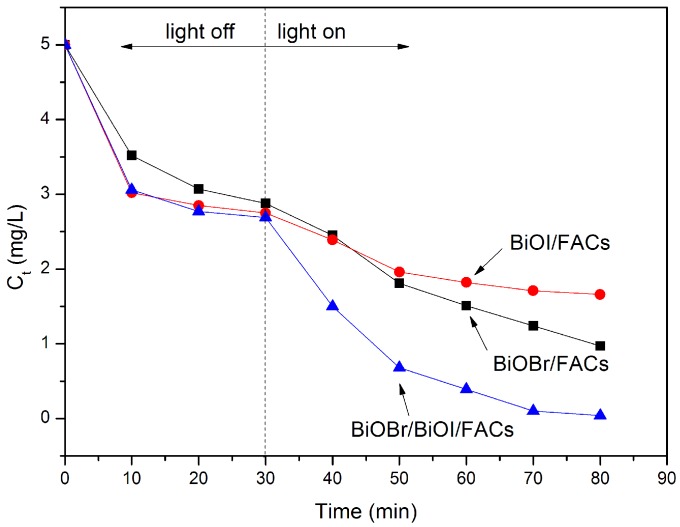
The degradation concentration-time curves of RhB over BiOBr/BiOI/FACs, BiOBr/FACs and BiOI/FACs.

**Figure 7:**
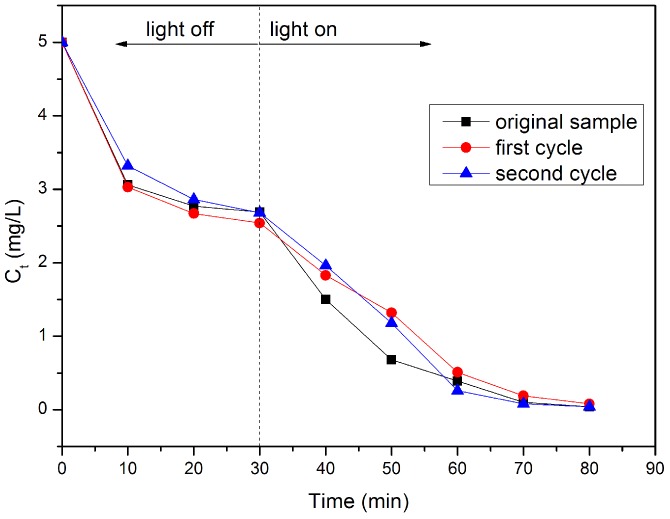
The concentration-time curves of RhB in BiOBr/BiOI/FACs photocatalysis cycles.

The visible light photocatalytic activities of the as-prepared photocatalysts in the degradation of RhB are further investigated as shown in Figure 5. Based on Figure 5, besides a little adsorption, the results indicated that the direct photolysis and photocatalytic degradation over pure FACs of RhB are negligible. However, after 30 min of dark equilibration, the percentage of RhB adsorbed on the surface of the BiOBr/BiOI/FACs reached 40%, 47%, 59% and 60%, respectively. This demonstrates that BiOBr/BiOI/FACs composites possess more outstanding adsorption ability than FACs, which basically agrees with the previous SEM and XRD analysis results. Subsequently, the removal efficiency of RhB increased under visible blue LED irradiation and more than 99% of RhB was removed in the following 30 min by the BiOBr/BiOI/FACs samples obtained at pH values of 9.0 and 11.0. This high efficiency degradation over BiOBr/BiOI/FACs may be attributed to the excellent absorption ability, more BiOBr/BiOI loaded on the surface of FACs and increasingly optical absorption in the visible light region (see Figure 2 and Figure 3) which are the critical factors in heterogeneous photocatalytic reactions [28,29]. These facts reveal that the alcoholysis pH value in the precursor suspensions for the coating of BiOBr/BiOI is a vital factor. Enhanced pH value-synthesized BiOBr/BiOI/FACs possess superior photocatalytic degradation activity for some organic comtaminants. For comparasion, BiOBr/BiOI/FACs, BiOBr/FACs and BiOI/FACs were prepared under the optimized conditions and the results of the corresponding photocatalytic activity tests are illustrated in Figure 6. Obviously, it can be seen that the photocatalytic activity of BiOBr/BiOI/FACs is higher than that of BiOBr/FACs and BiOI/FACs. According to [15], it is well known that photocatalysts with more positive VB edge potential possess stronger oxidative ability, so this could explain the higher visible light photocatalytic performance of pure BiOBr/FACs compared to pure BiOI/FACs in RhB degradation. In addition, it could be also speculated that BiOI and BiOBr could be all activated when BiOBr/BiOI composite is exposed under LED visible light irradiation (λ = 450 nm). The electrons in the VB of BiOI can be excited up to a further potential edge, then the photoinduced electrons on the surface of BiOI can migrate to the CB of BiOBr at the interface, and then reduce the adsorpted dissolved O_2_ to O_2_•^−^ due to the more negative CB edge potential than E° (O_2_/O_2_•^−^) (−0.13 eV NHE). The photoinduced holes can migrate from the VB of BiOBr to that of BiOI by the interface, as the VB edge potential of BiOBr is more positive than that of BiOI. Therefore, BiOBr/BiOI composites effectively reduce the recombination of photogenerated electrons and holes and improve the photocatalytic degradation efficiency. All in all this improved photocatalytic degradation performance over BiOBr/BiOI/FACs is attributed to the efficient separation of photogenerated carriers and the competition mechanism between the enhanced visible light absorption and the decreased VB edge potential [29]. In addition, the stability of the BiOBr/BiOI/FACs composite is also investigated in cycle experiments as shown in Figure 7. It can be seen that no significant activity loss was observed after a three cycle run. The result demonstrate that the composite photocatalyst exhibits stability in the process of RhB degradation.

## 3. Experimental Section

### 3.1. General Information

The phase purity and crystal structure of as-prepared samples are characterized by D/max-2550 X-ray diffractometry (Rigaku, Tokyo, Japan), Quanta-250 field emission scanning electron microscope (FEI, Hillsboro, TX, USA), energy dispersive X-ray spectroscopy (OXFORD ISIS, London, UK). The UV–Vis diffuse reflection spectra (DRS) are acquired with a UV-3100 UV–Vis spectrophotometer (Shimadzu, Kyoto, Japan) equipped with an integrating sphere, using BaSO_4_ as a reference. The X-ray photoelectron spectroscopy (XPS) measurement is carried out in PHI ESCA-5000C electron spectrometer (PE, Waltham, MI, USA). A TY-9800XP X-ray fluorescence spectrometer (XRF) (Wuxi jinyibo Instrument technology co., Wuxi, China) was employed for analyzing the chemical composition elements. According to the Table 2, the main chemical elements in fly-ash cenospheres are Al and Si.

### 3.2. Photocatalyst Synthesis

In a typical synthesis, Bi(NO_3_)_3_·5H_2_O (2.8 mmol) is dissolved completely in ethylene glycol (EG, 20 mL) with magnetic stirring at room temperature (25 °C). Then, KBr (1.4 mmol), KI (1.4 mmol) and FACs (2.0 g) are added into the beaker and dispersed by sonication for 30 min. Subsequently, the pH value of the mixture is adjusted to 5.0, 7.0, 9.0 and 11.0 by 35.0 wt % NH_4_OH solution and stirred for 30 min at room temperature. Afterwards, the mixture is filtered, collected, and maintained at 160 °C in an oven for 6 h. The obtained precipitate is washed several times with deionized water and dried at 65 °C for 6 h.

### 3.3. Photocatalysis Experiments

The photocatalytic experiments are carried out by using a 50 W blue LED lamp with a wavelength of 450 nm as the visible light source, which is placed about 5 cm from the surface of a liquid suspension of RhB. In each experiment, photocatalyst (0.1 g) was added to RhB solution (50 mL) with an initial concentration of 5 mg/L, The pH value didn’t change in reaction suspension system. Before irradiation, the suspension is stirred for 30 min in the dark to reach adsorption-desorption equilibrium. At given time intervals, approximately 2 mL of the suspension is filtered to remove the photocatalyst particles and the clarified solution is analyzed by UV–Vis spectroscopy.

## 4. Conclusions

Novel BiOBr/BiOI/FACs photocatalysts have been successfully fabricated through a facile one-pot alcoholysis approach. Based on the observations, more BiOBr/BiOI composites are loaded on the surface of FACs under alkaline conditions, giving higher photocatalytic properties under visible blue LED irradiation. The phocatalytic ability of BiOBr/BiOI/FACs was superior to that of BiOI/FACs and BiOBr/FACs and exhibited great stability and durability, still retaining over 90% degradation ability after several cycles. On the basis of the characterization results, we attribute the excellent photocatalytic activity to the presence of more active component loaded over the surface of FACs and effective electron-hole separation and transportation. More importantly, the novel composite, which shows excellent stablity, may have potential application in removing floating organic pollutants.

## Figures and Tables

**Table 1:** XPS quantification report of the as-synthesized BiOBr/BiOI/FACs samples.

Peak	Position BE (eV)	FWHM (eV)	R.S.F.	Area (CPS)	Atomic Conc%
O 1s	532.16	3.355	2.85	47588.3	81.3
Bi 4f	159.41	2.045	24.9	58683.4	11.47
Br 3d	68.66	2.211	3.04	3378.6	5.41
I 3d	619.56	1.897	32.7	12216.4	1.82

**Table 2:** Chemical composition of fly ash cenospheres.

Element	Fly Ash Cenospheres (wt %)
Na	1.46453
Mg	1.91019
Al	53.48339
Si	66.81443
P	0.15579
S	0.18619
K	0.24782
Ca	0.00320
Ti	0.37482
Fe	1.00285
